# How to approach and take care of minor adolescents whose situations raise ethical dilemmas? a position paper of the European academy of pediatrics

**DOI:** 10.3389/fped.2023.1120324

**Published:** 2023-06-07

**Authors:** Pierre-André Michaud, Yusuke-Leo Takeuchi, Artur Mazur, Adamos A. Hadjipanayis, Anne-Emmanuelle Ambresin

**Affiliations:** ^1^ University Hospital, Lausanne, Switzerland; ^2^ Division for Adolescent Health, Lausanne University Hospital and University of Lausanne, Lausanne, Switzerland; ^3^ Faculty of Medicine, University of Rzeszow, Rzeszow, Poland; ^4^ Medical School, European University of Cyprus, Nicosia, Cyprus

**Keywords:** young people, adolescent, ethics, decision making, health care, autonomy

## Abstract

In the care of adolescents, health care providers often face situations raising ethical concerns or dilemmas, such as refusal of a treatment or hospitalization, or request of confidentiality while engaging in risky behaviors or facing unplanned pregnancy. This position paper provides concrete avenues as how to assess the adolescent's capacity for autonomous decision making, e.g. the patient's competence in a specific situation, and how to elicit informed choice or consent. To do so, professionals need to be sensitized and trained as how to assess the cognitive and socio-psychological development of the young patient. Another challenge for the health professionals is to balance the needs to support patient's autonomy while offering secure guidance and protection if needed. To optimize such a process, they establish a climate of trust and empathy that will allow the patient to participate freely in the decision. In addition, especially when the decisions have potentially important consequences on the health and life, the professionals include, with the adolescent's permission, parents, caregivers or other significant adults, as well as they may request the opinion of other members of the health care team or expert colleagues such as ethicists.

## Introduction

Adolescents, defined by the World Health Organization (WHO) as individuals aged 10–19 years, undergo an important transition between childhood and adulthood (1, 2). This period is marked by a huge bio-psychosocial development which varies in pace from one individual to another (3).

*Maria is a fifteen years old adolescent who is brought to her school nurse because of recent recurring abdominal pain which impacts on her school functioning and grades. She is the only child of two parents who are heavily involved in their religious community and quite strict in Maria's education. Maria is doing fairly well at school but feels too controlled by her parents. She has begun to smoke e-cigarettes and occasionally uses cannabis. She has been drunk on one or two occasions, during her parents' professional trip outside the city. Her school grades have recently deteriorated. She is, since six month, dating with an eighteen years old boyfriend with whom she has–secretly - sexual intercourses. They use condoms often, but not consistently. Maria discloses to the nurse that she fears to be pregnant. The test performed by the nurse turns out to be negative, and Maria asks the nurse to keep the encounter confidential.*

This not uncommon situation raises concerns that many health professionals may face in their practice with adolescents: Should Maria be granted confidentiality or does this adolescent need more protection and should the parents know about their child's behaviour, despite the potential deterioration of the family life? Or is Maria cognitively and psychologically enough autonomous to manage her situation? What is the nurse's responsibility under these circumstances? Is it legally acceptable for a fifteen years old adolescent to date with a young adult who is 18? Does Maria need enforced education support to improve her school grades? In other terms, such situations raise psychological, legal and ethical issues that are unique to adolescents.

The adolescent brain undergoes vast structural and functional changes: this unique period of plasticity represents both a period of opportunity as well as potential risks (4, 5). Alongside with brain development, the level of cognitive, emotional and social maturation increases and the adolescent progressively develops the skills needed to understand the issues involved in decision-making. This notion of evolving capacity, for which age is not at all a sufficient indicator, implies an understanding of the need to promote the adolescents’ progressive participation in health care according to their level of maturity (6). Recent research has also shown that decision making capacity depends highly on the surrounding climate and the emotional state of the adolescent (7, 8). As stated by Steinberg (9), “Adolescents are indeed less mature than adults when making decisions under conditions that are characterized by emotional arousal and peer pressure, but many adolescents are just as mature as adults when emotional arousal is minimized and when they are not under the influence of peers, situations that typically characterize medical decision-making”. These authors thus refer to “hot” (arousal) vs. cold (calm) cognition (5). It is a particular challenge for health professionals to support adolescents in this participative process, as it requires multiple skills such as establishing a trustful relationship, offering an optimal setting to promote conditions for cold/peaceful cognition and evaluating their cognitive-psychological development. In addition, they have on one hand to support the young patient's autonomy and participation in decisions that affect health and well-being while, on the other hand, offering a setting that secures educational support and protection. Maria's situation illustrates the need to balance various ethical *values,* such as the individuals' rights to protection and autonomy, or the preservation of their dignity. It also raises the importance of legal and ethical principles.

The aim of this article is to review the ethical and legal aspects of such situations and to focus on how health professional should concretely handle them in their patients' best interest while involving stakeholders. The paper has been developed by members of the EAP (European Academy of Paediatrics) adolescent health strategic advisory group and two more experts in the field.

### Legal considerations and ethical principles as applied to adolescents

While the WHO defines adolescents as people aged 10–19 (1, 2), the United Nation Convention on the Rights of the Child (UN CRC), in its definition of children–including adolescents–sets a limit at 18 years, an age at which most countries also define the legal majority (6). This is of notable importance in the field of health, as individuals, from the age of their majority, are granted free autonomous decision making in all areas of health care. Since several decades, the UN CRC, with its 54 articles, offers a legal framework in health care, social services and education and, as such, has heavily impacted on how societies contribute to children and adolescents' autonomy in the decisions that concern their health and lives. It underlines the importance of the concepts of consent or assent and of protection. It stresses the fact that all decision pertaining to the child's education, development, well-being and health should be taken in *their best interes*t. A fundamental step in respecting their best interests is, as stated in the CRC, the right to participation, meaning the right to have their views expressed freely and those views given due weight in accordance with their developmental stage. Accordingly, minor adolescents are more and more considered as *partners* of health care professionals when it comes to making decisions regarding their health or the treatment of specific conditions. While some countries or regions set the age at which minor adolescents can exercise their rights in situations such as participating in clinical trial, experiencing sexual intercourse and using contraceptive methods, or being granted confidentiality, others do not establish fixed limits and thus rely on different stakeholders (including most of the time health care providers to determine whether the patient or subject is granted specific rights in various circumstances (10).

Since the creation of the Nuremberg code in 1947, several important documents have been developed that outline the foundation of biomedical ethics, such as the Helsinki declaration (1964) of the World Health Association or the Belmont Report which was developed in 1979 by the US National Commission for the Protection of Human Subjects of Biomedical and Behavioral Research (11) or more recently, the Council of Europe's European Convention for the protection of Human Rights and Dignity of the Human Being with regard to the Application of Biology and Medicine (1999) and even more recently, in 2016, the WMA Declaration of Taipei on Ethical Considerations regarding Health Databases and Biobanks. The 1979 Belmont Report (12) provides four essential principles - or moral values - that still lead health care professionals in addressing ethical issues. These principles were originally focused on how research on human subjects should be conducted but since then they also apply to the clinical care of all patients and to a large extent they affect preventive interventions. The four principles are the following:

• *Autonomy*: An autonomous person is person capable of reflexion about personal goals and of acting under the direction of such reflexion. Individuals with questionable autonomy should be entitled to protection.
• *Non maleficence*, (protecting patients from harm) echoes the Hippocratic principle known as the “primum not nocere” concept.
• *Beneficence*, reflects the stakeholder's obligation of: (1) not harm and (2) maximize potential benefits and minimize possible harms.
• *Justice/equity* ensures a fair distribution of social and medical resources, and stresses the issue of social equity, for instance having a voice for vulnerable population such as adolescents and children.

These principles perfectly illustrate the delicate balance between autonomous development and protection, as exemplified by Maria's situation: should this adolescent beneficiate from autonomous decision-making and benefit from confidential care as requested by her, e.g., the nurse not disclosing the situation to the parents? or would the adolescent need the guidance and support of her parents, which could be considered as beneficence and would imply a break of confidentiality, thus making the principle of protection prevail over that of autonomy. In paediatric settings, the concept of protection of minors still too often prevails at the expense of the principle of autonomy. The concept of beneficence is indeed often misinterpreted by many professionals, who tend to adopt a paternalistic approach and think that they themselves know what the best interest of the adolescent is, instead of gauging *with adolescents* what their best interest means for them. This is especially the case when the adolescent's opinion diverges from the one of their relatives or the one of the professional (13). In these situations, a tension may arise between the position of the professionals, based to some extent on scientific rationality but also on their personal values, and the position of young people based on their own experience and values (14, 15). It is therefore essential to avoid the assumption that adults “know better” than young people and to build a true partnership in which professionals accompany and support the deliberation and decision-making processes, while exploring the influence of their own personal values and representations. In Maria's situation, it means that the nurse, if accepting to keep the consultation confidential, should be confident that Maria in the future would be able to adopt more healthy and secure behaviour. There are obviously other principles or values that should guide ethical considerations, such as the individual's dignity and integrity (16). The principles pertain to all ethical issues, regardless of the age of the patient and are as such especially relevant for minor adolescents.

### Important prerequisites for managing ethical dilemma situations with adolescents

Three notions guide the application of ethical principles to the health of minor adolescents (17–19)

• *Competence* refers to a legal concept meaning that a person is able to understand the issues linked with a situation requiring a decision or/and to give an informed consent (13, 20, 21). In most regions of the world, young people 18 years and over are legally adults and as such considered competent, unless they suffer from a severe psychiatric disturbance or mental disability. In some regions or countries, minor adolescents are considered competent as long as, in a given situation, their health-care providers consider that they are capable of decision-making whereas in some instances, competence for specific situations is legally set at a defined age (13). While competence is a legal concept, health care providers usually prefer the notion of *autonomous decision-making capacity* which implies an evolving process and is linked to a specific time and a specific medical situation (19). In several European countries, such as Austria, Bulgaria, Norway, Ireland, Portugal, the Netherlands, Ukraine and Denmark, the right to receive information or to express one's will is granted according to a defined age limit. Interestingly this age limits varies a lot, between 7 years (Norway) to 16 (for instance. Bulgaria, Portugal) (22). In UK and Scotland, minor patients from 16 years of age or judged to be “Gillick competent” and are usually granted the right to consent to treatment but not to refuse it; these adolescents (<18 years) are often defined as “mature minors”. In other countries of Europe, the age at which young people have the right to receive information or to express one's will is not fixed by the law but rather based on the evaluation of the degree of maturity and the level of cognitive development (22). This is, to much extent, the case in Italy, Belgium, France, Germany, Finland, Hungary Switzerland and Monaco. As can be understood, the health care providers, in many countries need to evaluate their young patient's competence and whether they can make informed decision (see below).
• *Informed consent* covers a competent individual's right to make decisions about any health issue, such as a lab test, the prescription of a medication or of a surgery as well as the participation in a research or a preventive program (23, 24). Older children and very young adolescents who are not considered totally competent are invited to give their opinion regarding a health care procedure, and this is called an assent (17, 25). Still, professionals and parents are not required to follow the child's judgment, but should make every effort to take it into account. The use of the word “informed” is important, and stresses the fact that, to be able to give consent, the adolescent needs to be fully informed in a cognitively adequate and adapted language about all the medical and psychosocial aspects and consequences of the situation
• *Confidentiality* is defined as the right to request the non-disclosure of health information without the patient's consent (26, 27). In many regions of the world, confidentiality is ensured to any person considered competent, even before the age of legal majority. However, confidentiality must be broken in some situations, e.g. when someone is threatening their own or someone else's life, or in cases of physical or sexual abuse (28). Under these circumstances, the health care professional may be authorized or even legally compelled to break confidentiality according to the legal framework governing the protection of minors. In addition, even if the issue of confidentiality should be systematically raised, the health care provider should encourage adolescents to share as much information as possible with their parents, whose role as educators is crucial.

### How to assess and support an adolescent in his capacity for autonomous decision making (13, 18)

**Figure 1:**
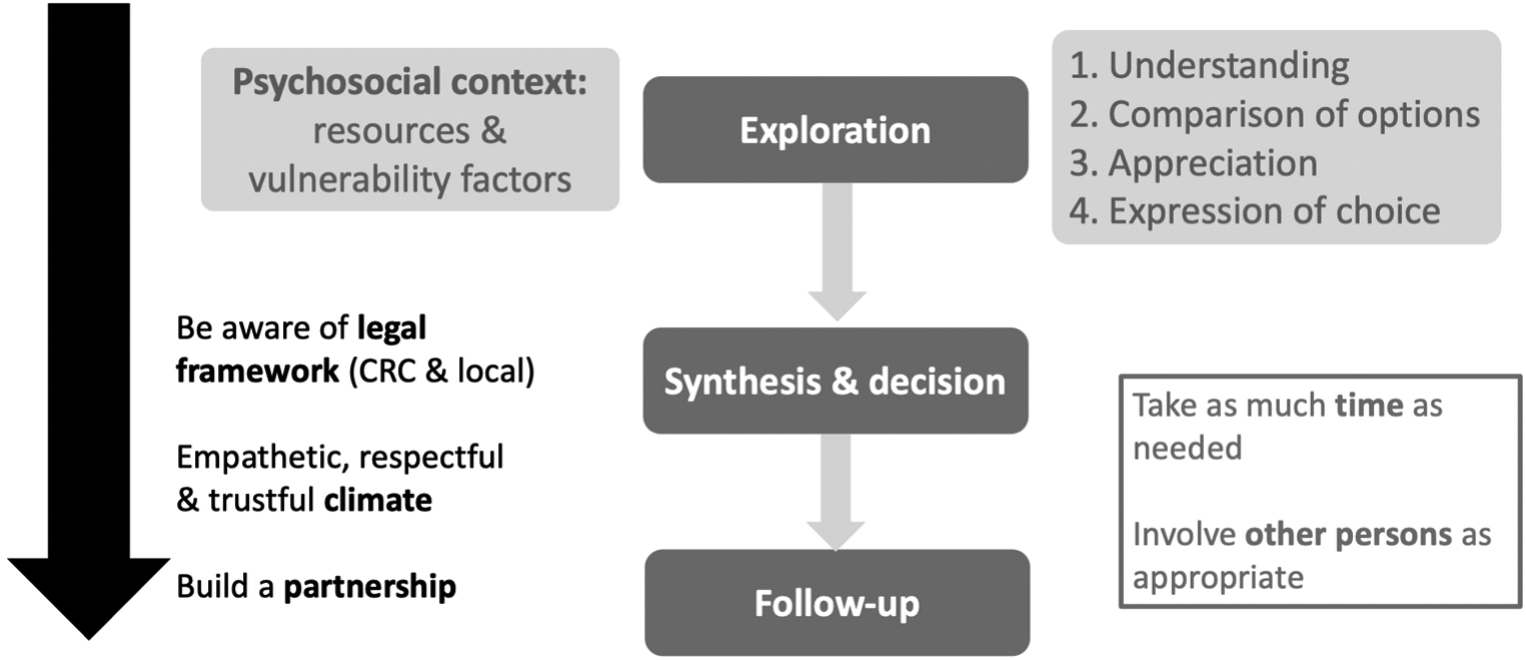
How to assess an adolescent's decision making capacity and address a situation raising ethical issues (adapted from ref 13).

In Maria's situation, can we consider that she is cognitively and psychologically capable to manage the issue of her contraception, or on the contrary, do we have reasons to doubt that she is fully competent, especially since she engages in several risky behaviours (smoking, drunkenness.)? In such situation, how can we best support Maria? This important and delicate process is summarized in Figure 1. First, the professional needs to set a proper framework of care and ensure a safe and empathetic climate, adapted to the specificity of the adolescent's development and situation. He will offer to meet the young patients alone for some time and explain to the parents–or foster parent - why the adolescent's confidentiality will be granted, even though open communication between them is encouraged. In some instances, the adolescent does not want to involve the parents at all, which can be justified e.g., if there is a risk of retaliatory measures. The professional can in this case attempt to involve another trusted adult, who will provide support to the young patient during the decision-making process. In addition, there is increasing evidence from neurodevelopmental research that the capacity to foresee long-term consequences of an option is not rarely hindered by the adolescent's emotional state (9). This means that ensuring optimal conditions for a good communication and mutual trust is a key factor for enhancing the autonomy in the decision-making process of adolescents.

The evaluation of autonomous decision-making capacity requires concrete field practice. First, the pace of cognitive and affective development varies among teenagers (29), which makes the definition of age cut-offs for autonomous decision making inaccurate. Moreover, competence varies by context and type of situation: it is not comparable to determine whether to check one's immunization status or to undertake an abortion for an unexpected pregnancy. Finally, while the assessment of an adult's competence, (e.g., when they suffer from a severe psychiatric disease or a cognitive deficit), can be completed with a structured interview instrument, such as the widely used MacCAT-T (30), it is problematic to rely on a standardized instrument for the evaluation of an adolescent's competence. Indeed, many factors underline the need for a more complex process to evaluate adolescents' capacity for decision making. Amongst them are the heterogeneity of adolescents' cognitive and psychological development stage at the same age, the influence of contextual factors and the fact that they are minors, which implies the obedience to specific legal frameworks and the necessity to take into account parents' views. WHO has recently developed a practical guidance for professionals in the field to assist them in the concrete process of assessing and supporting young people's competence, taking into account developmental, ethical and legal considerations (13). The document is particularly relevant for professionals working in countries which do not define an age limit for competence.

Among barriers to adolescents' participation in decision regarding, there is the professionals' underestimation of the adolescents' experience of health and diseases as well as their health literacy, especially if they suffer from a long-standing condition (7). In addition, some professionals are unaware of the importance of the setting, and of the key condition to establish a trustful relationship with the adolescents (31). Another barrier is the lack of training in specific skills to assess their stage of affective and cognitive development, when it comes to assessing decision making capacity. Last, there is a strong need to move from the old paternalistic approach we have been trained in towards a shared decision-making partnership. As a consequence, as stated by Alderson & al. “criteria for competence must move from age towards individual experience and understanding” (31): even older children, if considered as partners, reveal “hidden abilities”, a sound understanding of their situation. This applies especially to those who suffer from a chronic condition with which they have learned to live.

As a consequence, the assessment of the adolescent patient's capacity for decision-making should generally not be appointed to a specialist such as a psychotherapist or a lawyer, but should remain in the hand of a health professional who knows the adolescent's social and environmental context. This is not to dismiss the opinion of parents and other relatives or stakeholders who are familiar with the adolescent but the evaluation needs to be fulfilled with a high degree of objectivity, free of pressures. Additionally, the professional in charge of such assessments have to gauge the patients' mood and mental state, and check that anxiety, depression or even delusion do not modify their capacity to analyse and reflect. If the young person suffers from a disturbing psychological disorder or even a psychiatric disease, the opinion of a psychiatrist can be sought.

In summary, involving practitioners who know the adolescent well, seeking the opinions of parents, caregivers or other stakeholders, performing the evaluation in an empathetic, safe and peaceful atmosphere that enables a cold rather than hot cognition process are all prerequisites to support adolescent's decision-making capacity. This takes time and should be carried out ideally several times (18, 19, 32). In Maria's situation, the nurse could see the adolescent on more than one occasion to make sure that she understands all the facets and potential consequences of her situation and behaviour. The nurse could as well discuss the dilemma with her supervisor. Another tricky aspect of the situation is that Maria has engaged in sexual intercourse with a boyfriend who is three years older: in such circumstances, the nurse has to assess whether there is some power imbalance between the partners as well as Maria's capacity to have her needs and choices respected by her boyfriend; in addition, some legislations, while tolerating sexual intercourse between adolescents aged each 14 or 15, may still compel the nurse to disclose the situation to the parents and to the child protection authorities, due to the fact that the boy is fairly older and that this difference in age may potentially involve some pressure from the boy to engage in active sexual behaviour.

### Exploring with the adolescent the various facets of a situation raising ethical issues and values

*Tommy, a 15 years old adolescent has been suffering since several years from a severe leukaemia. The disease relapses despite several pharmacological treatments and a marrow graft. A final palliative treatment is offered, which could extend the child's life, but may involve heavy side effects. The adolescent is depressed with this situation and refuses the treatment, while the parents strongly insist on starting it.* As with an adult, the exploration of this situation covers a number of aspects. One is the concrete evaluation of how the adolescent appraises the situation. The professional is first invited to gauge whether Tommy understands his health condition and the related issue and options available to him; indeed, after years of treatment, he may assume that this is the case. The health care provider then reviews the adolescent's opinion and perception regarding the situation, the disease itself, its impact on his prognosis and on his daily life, whether undergoing the treatment or not. In this circumstances as well as in other problematic situations, questions can be asked such as: “what do you know and have learnt about your disease?”; “could you describe the effects of the treatment?”; “what would happen if you decide to engage in this treatment or if you decide not to?”; etc. Another domain to integrate in the evaluation is the psycho-social context. Assessing adolescents’ resources and risk factors is very important to draw the most comprehensive picture of the situation. These will be very important to weigh the decision when it comes to balance risks and benefits regarding the decision expressed by the adolescent. Some tools such as the HEADSSS guide can assist professionals in assessing the lifestyles and health behaviour of young patientst (33). In Tommy's cased, this is notably relevant, as he is depressed, which may potentially modify his perception of his situation.

In general, as expressed in the upper right part of Figure 1, the health professional should review how any young patient is able to reason, for instance in applying logic to refine information, confirm facts, validate change or persistence in opinions and beliefs established while acquiring new information (18, 19, 34). This can be achieved in asking questions, such as: “what would be the consequence of this procedure” or “are you informed of the potential difficulties linked with this treatment, and if yes, can you explain”; “what could happen..”; “now that you have described the risks of this option, how do you feel about it?”. The young patient is also asked to discuss the consequences linked with the different options, and balance their respective risks and benefits, with the support of the health professional. This is especially tricky when discussing with a young adolescent whose capacity to reason in abstract and develop time perspective is limited (35).

An important step is to explore how the young person perceives the relevance of the various options applied to their personal situation e.g., how they feel about their parents' or relative's views and preferences, and if these opinions could affect their decisions. In reviewing the different options that can be taken, the professional will try to figure out or balance the kind of *values* that are stressed by each of the options: some decisions emphasize the autonomy of the young person, while others clearly highlight a need for protection or still others may foster dignity, or equity (16). In Tommy's situation, the health care provider should anticipate the impact of the discontinuation of the treatment on his relationship with the parents and the life of the family. Obviously, while respecting Tommy's autonomy and maintaining the patient-centred perspective, the parents should be involved in the process and invited to share their concerns.

After such a careful, respectful deliberation, the adolescent is asked to clearly communicate the preferred choice and then to justify it in the light of the exchange which took place before. Some patients may be incapable to express a choice during the first encounter; they may feel too ambivalent or just need time to think about the issues. The young person may also be unable to overcome implicit or explicit pressure to make a certain choice, exerted by parents or even professionals. The professional should thus wait until he becomes confident that the patient has all the facets of the situation in mind and feels free top express his view. In Tommy's situation, ideally, the deliberation - which would take place on several occasions - would allow him, his parents and the health professional to agree with a decision accepted by all parties. If a consensus is not reached, a careful exploration of everyone's own values is important to see how they influence each point of view and to differentiate between a disagreement between values or decision-making capacity.

### A role for other stakeholders

Balancing the multiple ethical issues can be particularly challenging for professionals in complex situations with high emotional content in which a young person's decision may result in death like the one of Tommy. It can thus extremely useful to collect the standpoints of other relevant stakeholders, who can provide their own viewpoint to clarify the ethical issues related to the situation. Where possible, the involvement of a clinical ethic team to assist in the deliberative process may be advisable. Involving the parents can in some instances be problematic: on one side, the adolescent' autonomy and privacy has to be respected, but on the other side, the parents/the caregiver keep a right and also a responsibility to participate in the discussion. The professional has thus to make sure that the parents do not put too much pressure and ensure that the patients have sufficient time alone with them to reflect on their own before bringing parents in. In addition, if the parents diverge with the decision of their child and if this child is considered competent, the choice of the child should be respected (14), unless the law forbids it. In addition, there are other persons who can bring their own view under specific circumstances, such as a significant teacher, a social worker, a psychologist, or even in some instances a peer with significant role in the adolescent's life.

Lastly and most importantly, especially when it is not possible to obtain the stakeholders' opinions, the ultimate guiding principle for the physician faced with tough ethical decisions is to *not remain alone* and at least discuss the case with a trusted colleague, who may be less emotionally implicated. This is why working in multi-professional teams, or within a network of interdisciplinary collaboration is extremely useful, as each of the participants contributes with their own perspective. Many institutions or hospitals resort to regular formal inter-professional meetings to address situations with tough ethical aspects. Such meetings allow for a delicate consideration of the values linked with different options: What is beneficial for the patient? How can one preserve both self-government and dignity? How to secure an effective support of autonomy as well as a protection of the young patient? Balancing the judgment between various options can be better performed within a group discussion. When this proves not possible, the health professionals in charge of making the decision with the adolescent are advised to take time and not make any decision under rush. This will allow them to require an opinion of a colleague, reflect with some distance and come back with a more accurate evaluation.

### The adolescents' participation in research

The strategy described so far above applies to the adolescents' participation in biomedical research and is particularly relevant to clinical trials pertaining to new drugs or medical devices. In most legislation, children and adolescents are considered as “vulnerable” individuals and need a specific approach in any research including health information and data. It is beyond the objectives of this article to review all the facets of the involvement of minors in biomedical research. Suffice is to say that they should be, as far as possible, involved in the decision to participate and as fully informed as possible, taking into account their age and developmental stage (36–38). European countries do not provide a consensual vision of how to attain this goal (39): for instance, in Austria, individuals aged 15 years and over can make decision on their own, while in Finland, Denmark and UK the age is fixed at 16. Moreover, several countries have developed special access to consent depending on the type of research: in Switzerland for example, young people from age 14 are free to make their own decision if the risks linked with the trial is considered low. Even when they are not expected to give a consent in the legal context in which they live, they must be appropriately informed and invited to give an opinion (an “assent” under certain legal context), and their will respected if the situation is not life-threatening or heavily threatening their health and well-being.

One example of ethical aspects of research involving minors is the one of the participation of adolescents of Child Bearing Potential (40): the management of the issue of sexual development and behaviour is for many practitioners uncomfortable, given the young person's rights for confidential care. At what age and under which circumstances should the professional bring up the issue of contraception and sexual intercourse, given the high variation in pubertal timing? The research team must assess the obligation to impose regular pregnancy tests and contraception, which proves touchy when adolescents engage in active sexual intercourse without their parents knowing. Another issue is the one of questionnaires or surveys that tackle mental health: what is the role of the research team when adolescents disclose self-harm behaviour or former suicide attempt: what appropriate action should be taken in this case? How should, in these situation competence and decision-making capacity be assessed (41)? Are researchers equipped to conduct such evaluation? Such questions and issues should be adequately addressed within all paediatric research institutions.

There are additional ethical issues that cannot be explicitly tackled in this contribution for sake of space: one is the question whether to perform or not genetic tests or to disclose or not potential risks linked with genetic information. Other situations bring ethical dilemmas such as the refusal of hospitalization by adolescents facing a life-threatening condition.

### Recommendations of the European academy of paediatrics

A thorough step by step approach as described in this contribution is strongly recommended in every circumstance of ethical concern/dilemma in adolescents.

Health care professionals need to be trained in integrating the psychological and cognitive adolescent developmental stages in their care.

Fostering confidential care setting while building a trustful and empathetic relationship are essential conditions to assess and support the capacity for decision-making.

While parents should be part of the evaluation process most of the time, yet adolescents need to be seen alone for at least some part of the evaluation in order to promote and evaluate their decision-making capacity.

Adolescent's capacity for decision-making is an evolving process that need to be fostered by healthcare professionals from childhood onwards.

Faced with decisions especially difficult to make, health care providers should take time to reflect and, whenever possible, request the opinion of colleagues not emotionally involved.
